# Prevalence and predictors for spontaneous bacterial peritonitis in cirrhotic patients with ascites admitted at medical block in Korle-Bu Teaching Hospital, Ghana

**DOI:** 10.11604/pamj.2019.33.35.18029

**Published:** 2019-05-16

**Authors:** Amoako Duah, Kofi Nyaako Nkrumah

**Affiliations:** 1 Department of Medicine, St Dominic Hospital, Akwatia, Ghana; 2 Department of Medicine and Therapeutictics, School of Medicine and Dentistry, College Of Health Science, University Of Ghana, Korle-Bu, Accra, Ghana

**Keywords:** Liver cirrhosis, spontaneous bacterial peritonitis, ascites

## Abstract

**Introduction:**

Spontaneous bacterial peritonitis (SBP) is one of the most common and life-threatening complications of patients with cirrhotic ascites. Recognition and prompt treatment of this condition is essential to prevent serious morbidity and mortality. This study aimed to determine the prevalence of SBP among in-patients with cirrhotic ascites attending our facility and to determine the clinical and laboratory parameters associated with SBP.

**Methods:**

A cross-sectional study was conducted involving one hundred and three (103) patients admitted at medical block in the Korle-Bu Teaching Hospital (KBTH) with cirrhotic ascites from 25^th^ March, 2016 to 25^th^ November, 2016. Demographic and clinical data were collected using a standardized questionnaire. Ascitic fluid culture and cell count were conducted. Positive ascitic fluid culture and/or ascitic polymorphonuclear leukocyte ≥ 250cells/mm^3^ were diagnostic for SBP.

**Results:**

Of the 103 patients with cirrhotic ascites, the mean age was 43.5 ± 12.2 years. There were fifty eight (58) male patients. The prevalence of SBP was 25.24% (26/103). Majority, 5 (55.6%) of the bacteria isolated from ascitic fluid with SBP was Escherichia coli. Severe ascites and high INR were found to be independent predictors of SBP.

**Conclusion:**

SBP is common among patients with cirrhotic ascites admitted at KBTH. Severe ascites and high INR were highly suggestive of SBP. Diagnostic paracentesis should be done immediately on admission to confirm the diagnosis irrespective of the clinical characteristics as part of baseline investigation.

## Introduction

Spontaneous bacterial peritonitis (SBP) is a common and serious infection occurring in patients with cirrhosis and ascites [1]. Numerous studies suggest that 10-30% of hospitalized patients and 3.5% of outpatients with cirrhosis and ascites have SBP, with in-hospital mortality ranging from 20-40% [2-5]. SBP may be the precipitating factor for the occurrence of kidney failure, hepatic encephalopathy, gastrointestinal bleeding, hypervolemic hyponatremia and development of acute on chronic liver failure, systemic sepsis and poor survival [6]. An increase in the permeability of the intestinal wall leads to translocation of bacteria and subsequent development of SBP [7, 8]. Intestinal bacterial overgrowth and uncontrolled bacterial growth in ascitic fluid then occur, as a result of an impaired host immune response [9, 10]. Factors associated with the risk of developing SBP in cirrhotic patients include upper gastrointestinal bleeding, poor liver function, low ascitic fluid protein levels, prior SBP and hospitalization [11, 12]. Bacteria most commonly isolated from ascitic fluid in patients with SBP are usually those of the normal intestinal flora [13, 14]. More than 92% of all cases are monomicrobial with aerobic gram negative bacilli being responsible for more than two thirds of cases [13]. Escherichia coli accounts for nearly half of these cases followed by *Klebsiella* species and other gram negative bacteria. Twenty-five percent of cases are caused by gram positive organisms with *Streptococcus* species being the most common [13]. The symptoms and signs of SBP are subtle compared with those of patients who have surgical peritonitis in the absence of ascites. SBP may be asymptomatic in about 10-32% of cases, particularly in outpatients [4, 14, 15]. Symptoms and signs patients with SBP normally present with include fever, abdominal pain, altered mental status, abdominal tenderness, diarrhoea, paralytic ileus, hypotension and hypothermia (17%) [16]. Early diagnosis and prompt management of SBP once it has developed and preventing infections in high risk groups by giving prophylactic antibiotics are measures that can reduce morbidity and mortality in patients with liver cirrhosis. Because of the significant risk of adverse outcomes related to SBP, identifying predisposing factors is of utmost urgency. There are no recent published studies that exclusively address the prevalence and predictors of SBP in a hospital population in Ghana. This study aims to determine the prevalence and predictors of SBP among in-patients with cirrhotic ascites attending a tertiary referral center in Accra, Ghana. This will help with identification of high risk patients for early and appropriate treatment to reduce morbidity and mortality.

## Methods

A formal approval of this study was obtained from the Ethical and Protocol Committee of the University of Ghana School of Medicine and Dentistry. This study was conducted in accordance with the Helsinki Declaration. The research design was a cross -sectional hospital-based study, carried out at the Department of Medicine, Korle-Bu Teaching Hospital (KBTH), Accra, from 25^th^ March, 2016 to 25^th^ November, 2016. One hundred and three (103) patients with cirrhotic ascites admitted to the medical block of KBTH were consecutively recruited. All adult patients above 18 years with cirrhotic ascites who provided informed consent were included. Exclusion criteria were patients who had already been started on antibiotics at the time of recruitment or who had taken antibiotics up to 2 weeks preceding recruitment, as well as refusal of consent. Diagnosis of liver cirrhosis was made based on the clinical features, laboratory investigations and abdominal ultrasound findings suggestive of liver cirrhosis. Patients' medical records were reviewed to exclude those with cirrhotic ascites who were on antibiotics or had been on antibiotics in the preceding two weeks to the date of recruitment. Relevant history including alcohol use and clinical features of liver cirrhosis (spider angioma, palmar erythema, ascites, asterixis, hepatomegaly, splenomegaly and abdominal vein collaterals) were obtained. Ascites was graded as mild (detectable only on ultrasound), moderate (visible moderate symmetrical abdominal distension) or severe (marked abdominal distension). After thoroughly explaining the study to patients, individuals who gave informed consent were recruited and a questionnaire was administered (socio-demographic data and clinical history of the patients were obtained). A sample of 15mls of venous blood was taken for haematological, biochemical and serological investigations. Abdominal paracentesis was performed using an aseptic technique at the right or left iliac fossa, 3cm above and 3cm medial to the anterior superior iliac spine. Exactly 15mls of ascitic fluid was collected using a sterile syringe for culture, cell count and differentials, albumin and protein. Furthermore, an abdominal ultrasound scan was performed for all patients. The following details were recorded: maximum vertical span of the liver; nodularity of liver surface; spleen size (length of its longest axis); and presence of ascites.

**Ascitic fluid work up:** 10mls of ascitic fluid was inoculated into a blood culture bottle at the bed side and 5mls of ascitic fluid was inoculated into a sterile EDTA bottle for culture, cell count and differentials, albumin and protein. Ascitic fluid culture was performed by an experienced laboratory technologist by inoculating the ascitic fluid into Blood Agar and MacConckey Agar. Preliminary results were obtained after 48 hours, followed by conventional biochemical identification tests. SBP diagnosis was based on neutrophil count in ascitic fluid ≥ 250 cells/mm^3^ and/or positive ascitic fluid culture. If ascitic fluid cultures were positive and the neutrophils count was > 250cells/mm^3^, patients were diagnosed as having culture-positive neutrocytic ascites. If ascitic fluid cultures were negative in the presence of neutrophils > 250 cells/mm^3^, patients were characterized as having culture-negative neutrocytic ascites (CNNA). Patients with positive cultures on ascitic fluid but without neutrocytic ascites were classified as having monobacterial bacterascites (MNB).

**Blood test:** hematological, biochemical and serological workup included measurement of total serum bilirubin, serum albumin, alanine aminotransferase (ALT), alkaline phosphatase (ALP), gamma glutamyl transferase (GGT), aspartate aminotransferase (AST), serum creatinine, serum sodium (Na^+^), serum potassium (K^+^), heamoglobin (HB), platelet count, white blood cells (WBC), and prothrombin time with international normalized ratio (INR). Serological work up included hepatitis B surface antigen (HBSAg) and anti-bodies to hepatitis C virus (anti-HCV Ab). Anti-nuclear antibodies (ANA), immune globin G (IgG) and anti-smooth muscle antibodies (ASMA) were done for some of the patients with suspicion of autoimmune hepatitis. Model for end Stage Liver Disease Sodium (MELD-Na+) score was calculated based on laboratory parameters (bilirubin, creatinine levels, sodium and INR) collected at admission and determined by using the UNOS Internet site MELD-Na+ calculator. Child-Pugh Score (CPS) was calculated based on grade of ascites and hepatic encephalopathy, serum albumin, bilirubin and INR collected at admission using QxMD calculator on the net.

**Data analysis:** data obtained were analyzed using STATA 15 statistical software. Descriptive statistics was run for all the variables. The prevalence of SBP and other categorical variables were expressed as proportions. Biochemical parameters and the clinical scoring systems (MELD-Na^+^ Score and CPS) were reported as Mean ± SD (normal data) and median (IQR) (non-normal data). The Student t-test or Wilcoxon rank sum test were used to test the difference in means. Chi-squared test and the Fishers exact tests were used to determine the association of categorical variables and SBP. Univariable and multivariable logistic regression models were used to determine the strength of association between CPS, MELD-Na^+^ score, laboratory and other clinical parameters to SBP. This was presented as crude and adjusted odds ratios on a 95% confidence interval. For all analysis, p-values < 0.05 were considered statistically significant.

## Results

One hundred and three patients with cirrhotic ascites were recruited for the study with a mean age of 43.5 ± 12.2 years (age range 18 to 74) years. Fifty eight (58) (56.3%) patients were males and 44 (43.4%) were females with male to female ratio of 1.7:1. HBV infection was the cause of liver cirrhosis in 53.4% when acting alone and alcohol alone accounted for 21.4% of causes of liver cirrhosis. HCV infection was the cause in 8.9% of cases and HBV infection in combination with alcohol accounted for 6.9%. Autoimmune hepatitis, fatty liver disease and congenital atresia were uncommon causes (Table 1). The prevalence of SBP was 25.24% (95% CI=17.69-34.67). Of the 26 patients that had spontaneous bacterial peritonitis, culture positive SBP was present in 26.9% (9/26) while CNNA was found in 65.4% (17/26). The prevalence of MNB was 7.7% (2/26) in this study. The major isolates from the positive cultures were *Escherichia coli* (5/9, 55.6%) and *Klebsiella spp*. (2/9, 22.2%). *Staphylococcus epidermidis* and *Corynebacterium spp*. accounted for 1/9 (11.1%) each (Table 2). Results from the univariable logistic regression showed that patients who had SBP had increased odds of having severe ascites (cOR=4.20, 95% CI=1.52-11.66), fever (cOR=3.14, 95% CI=1.25-7.81), chills (cOR=4.42, 95% CI=1.61-10.55) and encephalopathy (cOR=3.03, 95% CI=1.16-7.92). There was a 4% reduced odds of SBP with increasing age (cOR=0.96, 95% CI=092- 0.99) (Table 3). The multiple logistic regression model was fitted whilst controlling for age, sex and the other variables in Table 3. The clinical feature significantly associated with SBP from the multivariable analysis was severe ascites (aOR=5.82, 95% CI=1.51-22.42) (Table 4). Laboratory parameters associated with SBP from the univariable analysis were INR (cOR=2.09, 95%CI=1.28-3.45), CPS (cOR=1.34, 95%CI=1.07-1.69) and MELD-Na^+^ score (cOR=1.09, 95%CI=1.03-1.16). The adjusted multivariable logistic regression model fitted using a stepwise approach (backward selection). The variables that remained in the final model were Fluid Albumin, Meld-Na^+^ Score and INR, though only INR (aOR=1.90, 95%CI=1.08-3.35) was a significant predictor of SBP (Table 5).

**Table 1:** Causes of liver cirrhosis

Aetiology	Number	Percentage
Hepatitis B virus	54	52.4
Alcohol	22	21.4
Hepatitis C virus	9	8.7
Alcohol + Hepatitis B virus	7	6.8
Autoimmune Hepatitis	4	3.9
Congenital Biliary Atresia	1	1.0
Unknown	6	5.8
TOTAL	103	100

**Table 2:** Bacteria isolated from ascitic fluid

Organism	Total number of isolates	Percentage
*Escherichia Coli*	5	55.6
*Corynebacterium spp*	1	11.1
*Klebsiella spp.*	2	22.2
*Staphylococcus epidermidis*	1	11.1
Total	9	100.0

**Table 3:** Bivariate analysis (Clinical and demographic parameters)

	SBP (N (%)		X^2^	p-value
Variables	ABSENT	PRESENT		
Age(yrs.)	**45.4± 11.6	39.1 ± 13.4	-	0.025^†^
**Sex**				
Male	43 (55.8)	15 (57.7)		
Female	34 (44.2)	11 (42.3)	0.027	0.870
**Ascites**				
Moderate	43(55.8)	6 (23.1)		
Severe	34 (44.2)	20 (76.9)	8.364	0.004^†^
**Jaundice**			0.541	0.462
Absent	36 (46.8)	10 (38.5)		
Present	41(53.3)	16 (61.5)		
**Abdominal pain**			0.387	0.534
Absent	38 (49.4)	11 (42.3)		
Present	39 (50.7)	15 (57.7)		
**Fever**			6.208	0.013^†^
Absent	51 (66.2)	10 (38.5)		
Present	26 (33.8)	16 (61.5)		
**Chills**			9.328	0.002^†^
Absent	60 (77.9)	12 (46.2)		
Present	17 (22.1)	14 (53.9)		
**Weight Loss**			0.217	0.642
Absent	15 (19.5)	4 (15.4)		
Present	62 (80.5)	22 (84.6)		
**Pedal edema**			0.481	0.488
Absent	20 (26.0)	5 (19.2)		
Present	57 (74.0)	21 (80.8)		
**Hematemesis**			1.038	0.310
Absent	65 (84.4)	24 (92.3)		
Present	12 (15.6)	2 (7.7)		
**Encephalopathy**			5.361	0.021^†^
Absent	62 (80.5)	15 (57.7)		
Present	15 (19.5)	11 (42.3)		

† - p < 0.05 – statistically significant X^2^- Chi-squared value

** - Mean (± SD)

**Table 4:** Univariate and multiple logistic regression (socio-demographic and clinical)

Variables	Crude OR (95%CI)	p-value	Adjusted OR (95%CI)	p- value
Age (years)	0.96 (0.92 - 0.99)	0.029^†^	0.97 (0.93 - 1.02)	0.286
Sex (Male)	1.07 (0.44 - 2.65)	0.87	2.57 (0.81 - 8.17)	0.11
Ascites (Severe)	4.20 (1.52 -11.66)	0.006^†^	5.82 (1.51 - 22.42)	0.011^†^
Fever (Present)	3.14 (1.25 - 7.81)	0.018^†^	4.29 (0.59 - 31.27)	0.151
Chills(Present)	4.42 (1.61- 10.55)	0.003^†^	1.35 (0.20 - 9.11)	0.756
Encephalopathy (Present)	3.03 (1.16 - 7.92)	0.029^†^	2.03 (0.66 - 6.20)	0.214

† - p < 0.05 – statistically significant

**Table 5:** Univariate and stepwise multiple logistic regression

Variables	Crude OR (95%CI)	p-value	Adjusted OR (95%CI)	p- value
Age (years)	0.96 (0.92 - 0.99)	0.029^†^	0.93 (0.81 - 1.07)	0.344
WBC	1.02 (0.98 - 1.01)	0.782	-	
GGT	0.996 (0.99 -1.01)	0.075	-	
INR	2.09 (1.28 –3.45)	0.003^†^	1.90 (1.08 – 3.35)	0.025^†^
CPS	1.34 (1.07 -1.69)	0.009^†^	0.98 (0.34 - 2.89)	0.982
MELD-Na+ Score	1.09 (1.03 –1.16)	0.002^†^	0.77 (0.48 – 1.24)	0.283

† - p < 0.05 – statistically significant

## Discussion

This study aimed to determine the prevalence, clinical and laboratory features predicting the presence of SBP among inpatients with cirrhotic ascites in a tertiary care center in Accra, Ghana. The prevalence of SBP in this study was 25.24%. This is similar compared to 10-30% found by most studies from the developed world [12,14, 17, 18] but lower than one reported by Oladimeji *et al.* [19]. This may be due to the severity of liver cirrhosis involved in the study. Oladimeji *et al.* [19] stated that almost all the patients recruited in their study were in Child's grade C compared with this study with majority of the participants in child's grade B and C The diversity of the clinical and laboratory parameters that is associated with the presence of SBP has been reported in various literatures. This justifies the indication for diagnostic paracentesis in all patients with decompensated cirrhosis with ascites admitted in the hospital. In a study by Evan's *et al.* [4] no clinical or laboratory parameters were found to be associated with the presence of SBP whiles Figueiredo *et al.* [20] identified serum albumin, complement C4 of ascitic fluid and upper gastrointestinal bleeding as independent predictors for the diagnosis of SBP. Hypoalbuminaemia, high MELD score, C-reactive protein, Child-Pugh stage C, low protein level in ascitic fluid, low prothrombin concentration, increased serum aspartate aminotransferase levels, high serum bilirubin, low platelet count, hepatic encephalopathy and abdominal pain have also been reported by other studies to predict SBP [21-24]. In this study fever, chills, hepatic encephalopathy, severe ascites, lower age group, high INR, high CPS and high MELD-Na+ predicted the presence of SBP. However, only severe ascites and high INR showed strong independent association with SBP from the multivariate analysis. Severe ascites and high INR are among five markers used to stage the severity of liver disease according to Child-Pugh rankings [25]. The higher the CPS, the greater the risk of SBP [5]. This helps to explain why 70% of cases of SBP are seen in patients with Child-Pugh class C cirrhosis [26]. In the current study, though the Child-Pugh score was not an independent predictor of SBP, but 80% of the patients with SBP were in Child-Pugh grade C.

HBV (52.4%) was the major cause of liver cirrhosis in this study followed by alcohol (21.4%). This compared similarly with other studies done in Africa [27-29] but differed from reports from the western countries [30, 31]. The most common causes of liver cirrhosis globally are thought to be HBV, HCV and alcohol but the causes vary from country to country and from region to region. In countries where alcohol consumption is more common, alcohol is the commonest cause of liver cirrhosis and in countries where chronic HBV infection is endemic, HBV is the commonest cause of liver cirrhosis [32]. HBV infection is endemic in sub-Saharan Africa including Ghana and this makes it the major cause of cirrhosis in this study. Alcohol abuse was the second commonest cause of liver cirrhosis in this study which implies that alcohol is a significant cause of liver cirrhosis in patients attending clinic at KBTH. This is of public health concern; therefore society should be educated on the harmful effects of alcohol abuse on the liver. In this study *E. coli* (55.6%) was the most common organism isolated followed by *Klebsiella spp* (22.2%). The isolation of these organisms is consistent with studies conducted by Bhuva *et al.* [33] and Oladimeji *et al.* [19] which shows *E. coli* as the dominant bacteria cultured in patients with spontaneous bacterial peritonitis.

## Conclusion

The common causes of decompensated liver cirrhosis with ascites admitted at Korle-Bu Teaching Hospital were chronic HBV and chromic alcohol abuse. Spontaneous bacterial peritonitis was common among patients with cirrhotic ascites admitted at Korle-Bu Teaching Hospital. Fever, chills, hepatic encephalopathy, severe ascites, high INR, high MELD-Na^+^ and CPS were predictors of SBP but only severe ascites and high INR were independent predictors. Therefore diagnostic paracentesis should be done immediately for all patients on admission to confirm the diagnosis of SBP preferably before starting empiric antibiotic therapy especially in patients with severe ascites and high INR.

### What is known about this topic

- There is diversity of clinical and laboratory parameters associated with the presence of SBP;
- Upper gastrointestinal bleeding is a risk factor associated with the risk of developing SBP.

### What this study adds

- Only severe ascites and high INR were independent predictors associated with SBP;
- Patient presented with upper gastrointestinal bleeding were not at high risk of developing SBP.

## Competing interests

The authors declare no competing interests.
